# Determination of the *Plasmodium vivax *relapse pattern in Camopi, French Guiana

**DOI:** 10.1186/1475-2875-8-278

**Published:** 2009-12-04

**Authors:** Matthieu Hanf, Aurélia Stéphani, Célia Basurko, Mathieu Nacher, Bernard Carme

**Affiliations:** 1 Centre d'Investigation Clinique - Epidémiologie Clinique Antilles Guyane CIC-EC Inserm 802, Centre Hospitalier de Cayenne, Guyane Française; 2 Laboratoire Hospitalo-Universitaire de Parasitologie et Mycologie Médicale, Equipe EA3593, UFR Médecine - Université des Antilles et de la Guyane, Cayenne, Guyane Française

## Abstract

**Background:**

Malaria is a major public health problem in French Guiana, where *Plasmodium vivax *has become the dominant malaria species since 2000. As in others endemic areas, it is important to specify the pattern of vivax malaria relapses and to try to discriminate efficiently re-infections from relapses.

**Methods:**

This study was conducted in children born between January 1, 2001 and December 31, 2008 in Camopi, an Amerindian village located in the Amazon forest (n = 325), using an open cohort design. Primary and secondary attack rates of *P. vivax *were calculated using survival analysis. With the difference between the primary and secondary rates, this study aimed to estimate indirectly *P. vivax *relapse rate and evaluate its time evolution.

**Results:**

Of the 1042 malaria attacks recorded, 689 (66%) were due to *P. vivax *(without mixed infection). One hundred and fifty one children had their primary attack with *P. vivax *and 106 had their two first attacks with *P. vivax*. In the absence of primaquine treatment, it was shown that *P. vivax *relapses mainly occurred during the first three months after the first attack. Thirty percent of children never had a relapse, 42% had a relapse before the first month after primary attack, 59% before the second month and 63% before the third month.

**Conclusion:**

This study confirmed that the relapse pattern in Camopi was compatible with the pattern described for the *P. vivax *Chesson (tropical) strain. In addition, due to the relapse rate time evolution, a simple arbitrary classification rule could be constructed: before 90 days after the primary attack, the secondary attack is a relapse; after 90 days, it is a re-infection. Adapted management of malaria cases based on these results could be devised.

## Background

Traditionally, *Plasmodium vivax *was considered as a relatively benign malaria parasite, which could be easily treated. For the past 10 years, this has been questioned [1]. It has been shown that malaria could be involved in severe disease [2]. Furthermore, *P. vivax *has recently re-emerged [3] and drug resistances, mainly chloroquino-resistance have developed in several areas[4]. Primaquine treatment, the only therapeutic option against relapse, might also be failing [5]. It requires a 14-day course and G6PD deficiency screening, which is difficult and costly in some areas.

In French Guiana, *P. vivax *has become the dominant malaria species since 2000, with more than 60% of malaria attacks [6]. Knowledge about its relapse pattern is limited. *Plasmodium falciparum *remains predominant on the Maroni River, where the maroon populations are mostly Duffy negative and, therefore, resistant to *P. vivax*. *Plasmodium malariae *has been observed in 2% of malaria cases.

Differentiation of re-infection from relapse is very difficult; most articles use a combined risk or rate of re-infection and relapse [7,8], or estimated the relapse risk based on the frequency of *P. vivax *during the non-transmission season [9,10]. Although molecular epidemiology introduced new methods to discriminate re-infection from relapse, these methods are very expensive and, in many cases, they could not address the question accurately [11-13]. In French Guiana, there is a widespread belief that *P. vivax *was of the 'tropical' type, which is characterized by an early primary attack, followed by a short latent period before appearance of frequent relapses during the next year, but no published evidence from French Guiana or elsewhere in South America supports this belief.

The study objectives were to determine the relapse pattern of *P. vivax *using an alternative method and to propose a simple classification to easily differentiate re-infections from relapses.

## Methods

This research was carried out in Camopi, an Amerindian village (Wayampi and Emerillon) located in the Amazon forest along the Oyapock River, which marks the eastern border with Brazil. The incidence of malaria there is around 40% [14]. A retrospective study using an open cohort design was carried out with all Camopi children born between January 1, 2001 and December 31, 2008.

The children came to the health center approximately once a month, generally at the time of illness or for systematic visits (i.e. vaccination). Every six months, there is a verification that all newborn children are included in the cohort, that all children are present in the village, and that all the malaria data is collected. Data for children for whom follow-up by the health center had been interrupted was right censored at the interruption date. It was assumed that all malaria attacks were recorded at the local health centre [15]. The Camopi health center ensures free early diagnosis and treatment of malaria cases. Because of the extreme isolation, no other health facilities (public or private) are available in this part of the Amazonian forest.

The list of all clinical malaria episodes, their date of occurrence, Plasmodium species identification were made in the Camopi health center by two physicians, two nurses, and one Amerindian assistant. All thin blood smears were first analysed in Camopi by a trained nurse and then checked by the parasitological unit of Cayenne General Hospital, the referent malaria center in French Guiana.

Malaria was defined as temperature > 38°C at the time of consultation or fever within the past 48 hours associated with Plasmodium asexual forms on a thin blood smear (detection threshhold: 50 *Plasmodium*/μl). During the study period, all confirmed cases of *P. vivax *received a three-day treatment of chloroquine (total 25 mg/kg). Cases with mixed infections and therapeutic treatment failure (attack in the two weeks following the first attack treated correctly) were excluded from the study. In Camopi, treatment with a combination of primaquine (14 days) and chloroquine (3 days) was initiated in 2005, but only for *P. vivax *cases following a first relapse, which did not affect analysis in this study. This prescription of primaquine implies respecting French prescription rules: systematic screening for G6PD deficiency (counter-indication if G6PD deficiency) and delivery as a nominal temporary use authorization.

All parents were given an explanation and written consent was obtained for the study. The study was reviewed and approved by the Ethical Committee of Antilles-Guyane. Two groups of patients were analysed. The first one included the whole population of Camopi children born between January 1, 2001 and December 31, 2008. It was assumed that the first *P. vivax *attack after birth in this group was due to a new infection. The second one included only those who had had a primary attack due to *P. vivax*. In this group, the secondary *P. vivax *attack occurring more than two weeks after a correctly treated primary attack was due to either re-infection or relapse. Therefore, it was assumed that the difference between primary and secondary attack rate of *P. vivax *could be an estimate of the relapse rate. According to the above reasoning, the yearly incidences and the monthly rates of attacks in these groups were estimated using survival analysis methods. Data were right censored by time of child departure from village or death, or time to attack if attack was due to another Plasmodium species. Monthly attack rates were calculated by derivation of the survival curves. Monthly relapse rates were then calculated by difference between the secondary and primary attack rates. All statistical analyses were conducted using R 2.7.1 and the package Epicalc [16].

## Results

Between January 2001 and December 2008, 1,042 malaria attacks were recorded in Camopi in 325 children born during this period, of which 319 (31%) were due to *P. falciparum*, 689 (66%) to *P. vivax*, 21 (2%) to mixed infections and 13 (1%) to *P. malariae *or *Plasmodium sp*. One hundred and fifty one persons had their primary attack with *P. vivax *and 106 had their two first attacks with *P. vivax*. Annual incidence and survival time in the two groups were synthesised in table 1. Under the working hypothesis 0.12 relapses occurred each year per person who had had a first attack caused by *P. vivax*. Using incidences of each group, the proportion of relapses in the whole secondary *P. vivax *attacks was estimated at 28%.

**Table 1:** Annual incidence rate^$^* and survival time of the primary and secondary *P. vivax *attack in the Camopi children population.

	STUDIED POPULATION	NUMBER OF SUBJECTS	TIME AT RISK	INCIDENCE RATE	SURVIVAL TIME (YEAR)			RELAPSE INCIDENCE RATE
					25%	50%	75%	
**PRIMARY *P. VIVAX *ATTACKS**	The Camopi Children population	325	571.76	0.31	1.23	2.33	4	0
**SECONDARY *P. VIVAX *ATTACKS**	Children having had a primary *P. Vivax *attack	151	100.42	0.43	0.76	2.14	3.58	0.12

^$^: Rate unit: attack/persons at risk/per year

*: Right censoring was used in survival analysis

**Figure 1:**
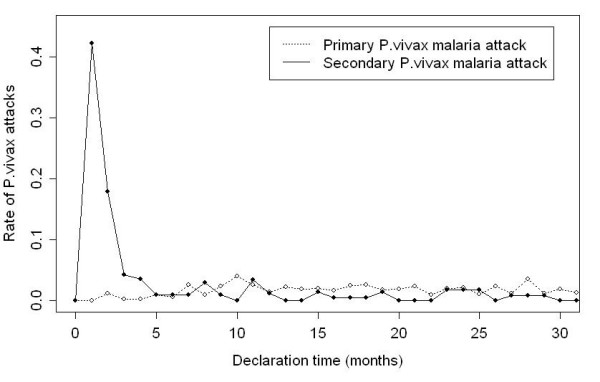
**Monthly attack rates^$ ^since birth for the primary *P. vivax *malaria attacks and monthly attack rates^$ ^since the first attack for secondary *P. vivax *attacks**. ^$ ^Rate unit: attack/100 persons at risk/per month.

Figure 1 compares the monthly rate of first *P. vivax *attack since birth and the monthly rate of secondary attack since first *P. vivax *attack classified by the time to attack. The pattern of changes in both groups was clearly distinct. The rate of primary attack seemed to be constant and uniform along the time and was approximately 0.03% per month and 100 persons at risk. On the contrary, the rate of secondary attacks had a sharp peak during the three first months following the first attack and reached a uniform and constant level after four months comparable to the rate of primary attacks. When relapse rate temporal trends were computed according to the working hypothesis, *P. vivax *relapses were shown to mainly occur during the first three months after the primary attack. A survival curve for relapses was computed. Thirty percent of children never had a relapse, 42% had a relapse before the first month after the primary attack, 59% before the second month and 63% before the third month. After eight months the probability to relapse was nil.

## Discussion

This study showed that, in Camopi, the peak of relapses of *P. vivax *occurred in the first three months after the first attack. After that, most secondary attacks were mainly due to a new *P. vivax *re-infection. These observations confirmed the assumption of a pure tropical pattern with a short latent period for *P. vivax *relapses in French Guiana (Chesson strain [17]). In India, the existence of both tropical and temperate zone types of *P. vivax *characterized by distinct incubation period for relapse were clearly suggested [9].

The current biological methods to differentiate re-infections from relapses have their limits. This study suggests that this simple alternative can estimate the relapse rate in *P. vivax *infection indirectly. However, this method has its own limitations. First, this method to estimate the relapse rate cannot be used in areas where primaquine is associated to chloroquine to treat the first and the second malaria attacks.

Second, it cannot differentiate the relapses from re-infections in individual patients; it estimates the overall relapse rate in the population. It seems that this limitation can be partially resolved by a simple arbitrary classification rule inferred from the results ofthis study. At an individual scale, before 90 days there is a strong probability that the secondary attack is a relapse, after 90 days it is more probable that the secondary attack is a re-infection. By positing that the primary rate of infection is the same than the secondary rate of re-infection, it can be assumed that immunity against *P. vivax *after the primary attack is negligible. As shown elsewhere [18,19], this does not seem to be an unreasonable hypothesis.

Third, similarly, because this study was based on the follow-up of children from birth, the re-infection rate based on the primary attack rate was under-estimated because of the maternal protective immunity during the first 12 months after birth. However, this effect plays in the opposite way of the previous bias and it didn't seem to influence significantly study results.

## Conclusion

This study confirmed that the relapse pattern in Camopi was compatible with the pattern observed with the *P. vivax *Chesson (tropical) strain. In addition, due to the relapse rate time evolution, a simple arbitrary classification rule could be constructed: before 90 days after the primary attack, the secondary attack is a relapse; after 90 days it is a re-infection. Adapted management of malaria cases based on these results could be devised: systematic prescription of primaquine for all relapses defined by this three-month rule, despite the obligations of screening for G6PD deficiency and administrative formalities involved on French territories. These results could be used in French Guiana and elsewhere in South America to discriminate *P. vivax *relapses from re-infections.

## Competing interests

The authors declare that they have no competing interests.

## Authors' contributions

MH participated in the research design, performed data analysis and interpretation, and prepared the manuscript. AS was responsible for data collection and participated in manuscript revision. CB participated in data analysis and manuscript revision as well. MN provided guidance on data analyses and was involved in the interpretation of data and manuscript revision. BC has designed the cohort and initiated the study, and was involved in the interpretation of data and manuscript revision. All authors read and approved the final manuscript
